# Health-related quality of life of patients of Brazilian primary health care

**DOI:** 10.11606/S1518-8787.2017051007134

**Published:** 2017-09-22

**Authors:** Bruna de Oliveira Ascef, João Paulo Amaral Haddad, Juliana Álvares, Augusto Afonso Guerra, Ediná Alves Costa, Francisco de Assis Acurcio, Ione Aquemi Guibu, Karen Sarmento Costa, Margô Gomes de Oliveira Karnikowski, Orlando Mario Soeiro, Silvana Nair Leite, Micheline Rosa Silveira

**Affiliations:** I Programa de Pós-Graduação em Medicamentos e Assistência Farmacêutica. Faculdade de Farmácia. Universidade Federal de Minas Gerais. Belo Horizonte, MG, Brasil; II Departamento de Medicina Veterinária Preventiva. Universidade Federal de Minas Gerais. Belo Horizonte, MG, Brasil; III Departamento de Farmácia Social. Faculdade de Farmácia. Universidade Federal de Minas Gerais. Belo Horizonte, MG, Brasil; IV Instituto de Saúde Coletiva. Universidade Federal da Bahia. Salvador, BA, Brasil; V Faculdade de Ciências Médicas. Santa Casa de São Paulo. São Paulo, SP, Brasil; VI Núcleo de Estudos de Políticas Públicas. Programa de Pós-Graduação em Saúde Coletiva. Universidade Estadual de Campinas. Campinas, SP, Brasil; VII Programa de Pós-Graduação em Saúde Coletiva. Departamento de Saúde Coletiva. Faculdade de Ciências Médicas. Universidade Estadual de Campinas. Campinas, SP, Brasil; VIII Programa de Pós-Graduação em Epidemiologia. Faculdade de Medicina. Universidade Federal do Rio Grande do Sul. Porto Alegre, RS, Brasil; IX Faculdade de Ceilândia. Universidade de Brasília. Brasília, DF, Brasil; X Faculdade de Ciências Farmacêuticas. Pontifícia Universidade Católica de Campinas. Campinas, SP, Brasil; XI Departamento de Ciências Farmacêuticas. Universidade Federal de Santa Catarina. Florianópolis, SC, Brasil

**Keywords:** Patient Satisfaction, Quality of Life, Pharmaceutical Services, Primary Health Care, Health Services Research, Unified Health System, Satisfação do Paciente, Qualidade de Vida, Assistência Farmacêutica, Atenção Primária à Saúde, Pesquisa sobre Serviços de Saúde, Sistema Único de Saúde

## Abstract

**OBJECTIVE:**

To analyze the Health-Related Quality of Life (HRQoL) of patients of the primary health care of the Brazilian Unified Health System (SUS) and its associated factors.

**METHODS:**

This is a cross-sectional study with data from the *Pesquisa Nacional sobre Acesso, Utilização e Promoção do Uso Racional de Medicamentos – Serviços, 2015* (PNAUM – National Survey on Access, Use and Promotion of Rational Use of Medicines – Services, 2015). Data were collected with a questionnaire that included the *EuroQol 5 Dimensions* (EQ-5D) instrument. Patients from the five regions of Brazil were interviewed. Multiple linear regression was used to analyze their Health-Related Quality of Life and its associated factors.

**RESULTS:**

Of the total of 8,590 patients, the most frequent dimensions were pain/discomfort (50.7%) and anxiety/depression (38.8%). About 10% of the patients reported extreme problems in these dimensions. The following factors were significantly associated with a worse quality of life: being female; having arthritis, osteoarthritis, or rheumatism; cerebrovascular accident; heart disease; depression; health self-assessment as poor or very poor; drinking alcoholic beverages once or more per month; dieting to lose weight, avoiding salt consumption, and reducing fat intake. Significant association was observed between a better quality of life and: living in the North and Southeast regions of Brazil; practicing physical activities; and having a higher educational level. No association was observed with factors related to the health services.

**CONCLUSIONS:**

The Health-Related Quality of Life of patients was influenced by demographic and socioeconomic factors that were related to health conditions and lifestyle, being useful to guide specific actions for promoting health and the integral care to patients of the Brazilian Unified Health System.

## RESUMO

**OBJETIVO:**

Analisar a qualidade de vida relacionada à saúde dos usuários da atenção primária em saúde do Sistema Único de Saúde e fatores a ela associados.

**MÉTODOS:**

Estudo transversal com dados da Pesquisa Nacional sobre Acesso, Utilização e Promoção do Uso Racional de Medicamentos – Serviços, 2015. A coleta de dados foi por meio de questionário que incluiu o instrumento *Euroqol 5 Dimensions*. Foram entrevistados usuários das cinco regiões do Brasil. Regressão linear múltipla foi utilizada para analisar a qualidade de vida relacionada à saúde e fatores associados.

**RESULTADOS:**

Do total de 8.590 usuários, as dimensões com maior frequência foram dor/mal-estar (50,7%) e ansiedade/depressão (38,8%). Cerca de 10% dos usuários reportaram problemas extremos nessas dimensões. Os fatores significantemente associados à pior qualidade de vida foram: ser do sexo feminino; ter artrite, artrose ou reumatismo; acidente vascular encefálico; doenças do coração; depressão; relatar autopercepção de saúde ruim/muito ruim; usar bebida alcoólica uma vez ou mais por mês; fazer dietas para perder peso, evitar o consumo de sal e reduzir o consumo de gordura. Foi observada associação significante entre uma melhor qualidade de vida e: residir no Norte e Sudeste; praticar atividades físicas e nível educacional mais alto. Não foi observada associação com fatores relacionados aos serviços de saúde.

**CONCLUSÕES:**

A qualidade de vida relacionada à saúde dos usuários foi influenciada por fatores demográficos, socioeconômicos, relacionados às condições de saúde e ao estilo de vida, sendo útil para nortear ações específicas de promoção da saúde e cuidado integral à saúde dos usuários do Sistema Único de Saúde.

## INTRODUCTION

Quality of life is an important measure of impact on health, being considered also an instrument for the promotion of health^5^ ^,^ ^12^. The measuring of the Health-Related Quality of Life (HRQoL) refers to how individuals evaluate their own overall well-being and health^7^.

Various instruments are available to measure the HRQoL, among them the *EuroQol 5 Dimensions* (EQ-5D) instrument. Simple, short, and easy to use, the EQ-5D has applications in the clinical and economic evaluation of health care, as well as in health research in populations^24^. The EQ-5D is a generic instrument that generates not only a health profile, but also an index that expresses the HRQoL of the interviewed individuals^9^.

International studies with the EQ-5D show that the HRQoL can be influenced by sex, age group, income, chronic conditions, as well as access and use of health services^1^ ^,^ ^18^ ^,^ ^22^ ^,^ ^26^. In Brazil, a research^17^ performed with the EQ-5D in the state of Minas Gerais, with 3,363 literate individuals, ranging from 18 to 64 years old, pointed out the presence of significant inequities in health. Older adults, women, and individuals with poor health and socioeconomic conditions showed more health problems^17^. However, in Brazil, studies with the EQ-5D on the HRQoL of the patients of the Brazilian Unified Health System (SUS) were not found.

With the basic rule of not only adding years to life, but also life to years, the measurement of the HRQoL and its associated factors are fundamental in the seeking of better living conditions for the populations^4^ ^,^ ^14^.

The *Pesquisa Nacional sobre Acesso, Utilização e Promoção do Uso Racional de Medicamentos – Serviços* (PNAUM – National Survey on Access, Use and Promotion of Rational Use of Medicines – Services) aimed at characterizing the organization of pharmaceutical services in the primary health care of SUS, to promote the access and rational use of medicines, as well as to identify and discuss the factors that affect the consolidation of pharmaceutical services in the cities.

This study is part of PNAUM – Services and aims to analyze the HRQoL of patients of the primary health care of SUS and its associated factors.

## METHODS

This study is part of PNAUM, a cross-sectional, exploratory, evaluative study, consisting of an information gathering in a representative sample of primary health care services in cities of the five Brazilian regions. Several populations were considered in the sampling, with samples having been stratified by the regions that constitute the study’s domains. In-person interviews were conducted with patients, physicians, and professionals responsible for the dispensing of medicines in the primary health care services of SUS. In addition, we observed the conditions of the pharmaceutical services’ facilities and conducted telephone interviews with the professionals responsible for pharmaceutical services in the cities. The pilot test was performed and standardized training was conducted with all interviewers for the in-person step. In this study, data concerning the interviews with patients of the primary care of SUS were used. The sample size was set to 1,800 patients by region of the Country. Considering the occurrence of a non-response percentage of 15%, 2,100 patients were randomly selected. Patients of SUS who were older than 18 years, waiting for a doctor’s appointment at the primary care service, able to answer the questions proposed, and who agreed to participate in the research were included. Patients were selected at random by the interviewers. It was established that the user chosen would be the last patient to be seen by the doctor among those who were already present in the unit. The data were collected between July and December 2014.

The PNAUM – Services methodology, as well as the sampling process, are described in detail by Álvares et al.^2^.

For the measurement of the HRQoL, the EQ-5D-3L instrument was used, which is composed of a descriptive system that encompasses five dimensions (mobility, self-care, usual activities, pain/discomfort, and anxiety/depression) with three levels in each (no problem, moderate problems, and extreme problems). Health condition is defined by combining one level of each of the five dimensions, being represented by a five-digit number. Thus, the EQ-5D-3L system defines 243 possible health conditions. Each health condition generated can be converted into a single score or index of the EQ-5D-3L, which incorporates the social preferences for the health conditions^26^. The EQ-5D-3L was validated in the Brazilian population. Therefore, the utility values obtained via the time trade-off technique by the QALY Brasil group were adopted to represent the unique health preferences of the Brazilian population, ranging from 1 to −0.176^25^. The EQ-Visual Analogue Scale was not used.

For the analysis of the factors associated with the HRQoL, the independent variables were sorted into four groups: i) demographic: sex, age group, skin color (white or non-white – black, yellow, mixed race, and indigenous), and region of residence; ii) socioeconomic: education level and socioeconomic class, according to the criteria of the *Associação Brasileira de Empresas de Pesquisa* (ABEP – Brazilian Association of Research Enterprises)^a^, 2013; iii) related to health conditions and lifestyle: self-reported chronic conditions, such as hypertension, *diabetes mellitus*, heart disease, dyslipidemia (high cholesterol and/or triglycerides), personal history of cerebrovascular accident (CVA), chronic lung disease (asthma, chronic bronchitis, emphysema, or other); arthritis, osteoarthritis, or rheumatism and depression; frequency of use of alcoholic beverages (never, less than once a month, and once or more per month); practice of physical activities in the past three months; being a current smoker; dieting to avoid the consumption of salt; dieting to lose weight; dieting to reduce fat intake; dieting to reduce sugar intake; self-assessment of health; and use of medicines in the 30 days prior to the interview); and iv) related to health services: having a health care plan or insurance; having used the emergency service in the 12 months prior to the interview; having been admitted to a hospital in the 12 months prior to the interview.

For the description of the variables, tables of distribution of frequencies for categorical variables and of means and standard deviation for numeric variables were made, with specific estimates and 95% confidence intervals. To verify the association between the EQ-5D-3L index and the independent variables, firstly a simple linear regression adjusted by region was performed. All variables with p<0.20 in the analysis of association with the HRQoL were included in the multiple model. The joint effect of the independent variables on the EQ-5D-3L index was evaluated by using multiple linear regression. The adequacy of the model was evaluated using the analysis of residues. A significance level of 5% was adopted. The data were analyzed using the STATA^®^ software, version 12.0.

PNAUM was approved by the National Research Ethics Committee of the National Health Council, under Opinion no. 398,131/2013. All participants signed the informed consent form.

## RESULTS

PNAUM – Services carried out interviews with 8,803 patients from 1,305 primary health care services, located in 272 cities distributed in the five geopolitical regions of Brazil. 213 patients were excluded due to the absence of complete questions of the EQ-5D-3L or for lack of data. Thus, the total sample of this study included 8,590 (97.5%) patients who reported their HRQoL, from 1,139 health services.

Table 1 shows the characteristics of patients of the primary health care of SUS who answered questions about their HRQoL.

**Table 1:** Characteristics of patients of the primary health care of the Brazilian Unified Health System. National Survey on Access, Use and Promotion of Rational Use of Medicines – Services, 2015. (n = 8,590)

Variable	n^a^	%^b^
DEMOGRAPHIC		
Sex		
Female	6,617	76.1
Male	1,973	23.9
Age group (years)		
18 to 39	3,062	39.7
40 to 59	3,054	37.3
60 or more	1,805	23.0
Skin color		
White	3,072	40.0
Non-white	5,518	60.0
Region		
Midwest	15,116	5.9
North	1,544	5.5
Northeast	1,681	29.9
South	2,019	24.9
Southeast	1,830	33.9
SOCIOECONOMIC		
Education level		
Illiterate	736	10.1
Some Elementary or Middle School	3,192	40.8
Elementary or Middle School	1,870	20.4
High School	2,499	25.6
Higher Education	293	3.1
Economic class		
A/B	1,389	14.9
C	5,046	55.1
D/E	2,155	30.0
HEALTH CONDITION AND LIFESTYLE		
Hypertension		
Yes	3,071	38.6
*Diabetes mellitus*		
Yes	1,121	13.6
Heart diseases		
Yes	624	7.8
Dyslipidemia		
Yes	1,901	22.9
Cerebrovascular accident		
Yes	211	2.5
Chronic lung disease		
Yes	871	9.6
Arthritis/osteoarthritis/rheumatism		
Yes	1,629	19.6
Depression		
Yes	1,490	18.5
Practice of physical activity		
Yes	2,313	26.1
Smoker		
Yes	1,084	13.3
Diet to lose weight		
Yes	1,669	18.5
Alcohol intake		
Never	6,456	76.3
Less than once a month	1,061	11.9
Once or more per month	1,072	11.8
Avoids salt intake		
Yes	5,239	59.2
Diet to reduce fat intake		
Yes	4,821	54.7
Diet to reduce sugar intake		
Yes	4,001	44.0
Self-assessment of health		
Very good/good	4,961	57.1
Neither good nor poor	2,957	35.0
Poor/very poor	664	7.9
Use of medicines		
Yes	6,506	76.6
HEALTH SERVICE		
Health insurance		
Yes	738	9.8
Used emergency service in the past year		
Yes	2,142	22.9
Was admitted to a hospital in the past year		
Yes	832	9.6

^a^ Non-weighted *n* value

^b^ weighted %

Source: PNAUM – Services, 2015.

The five most prevalent conditions were hypertension (38.6%), dyslipidemia (22.9%), arthritis, osteoarthritis, or rheumatism (19.6%), diabetes (13.6%), and depression (18.5%). More than 70% of the patients declared not having the habit of consuming alcohol, smoking, practicing physical activities, or dieting. More than half of the patients declared avoiding the consumption of salt and fat and about 45% reported avoiding sugar consumption (Table 1). Among the patients who reported dieting (n = 7,117), 39.4% declared to be following medical advice or advice from a nutritionist, 45.1% to be doing it on their own, and 15.5% for another reason or not knowing why.

Most patients (76.6%) claimed to have used medicines in the past 30 days. Regarding the self-assessment of health, 57% evaluated their health as very good or good. Almost 90% of the patients reported not having health insurance. Most patients declared not having been admitted to hospitals (77%) or having used the emergency service (90.2%) in the year preceding the interview (Table 1).

According to the descriptive system of the EQ-5D-3L, 115 health conditions were identified in the population studied, among the 243 possible ones. The ten most prevalent health conditions correspond to 81% of the population. The most common health condition was perfect health (11111), which represented 36% of the patients interviewed. The worst health status (33333), with extreme problems in all dimensions, corresponds to 0.01% of the sample.

**Figure 1:**
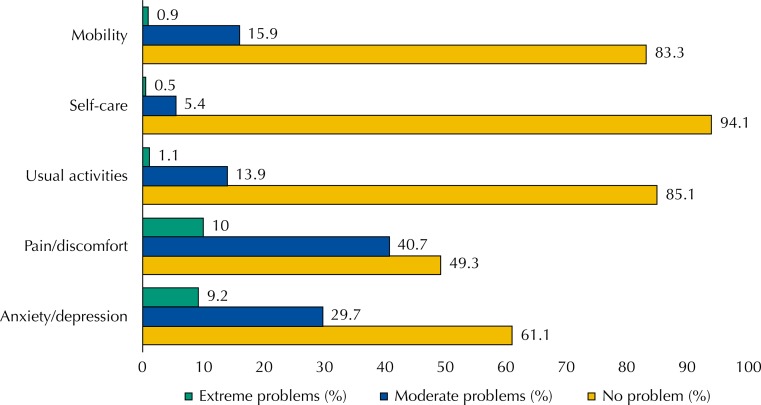
Percentage of patients of the primary health care of the Brazilian Unified Health System by level of problems reported for each dimension of the EQ-5D-3L in Brazil. National Survey on Access, Use and Promotion of Rational Use of Medicines – Services, 2015. (n=8,590)

Source: PNAUM – Services, 2015.

Figure 1 presents the percentages of patients of the primary health care in Brazil, by level of reported problems, for each dimension of the EQ-5D-3L. More than 80% of the patients reported problems in mobility, self-care, and usual activities. About 15% of the patients declared moderate problems in mobility and in usual activities and only 5.4% reported moderate problems with self-care. The percentage of patients with extreme problems in these dimensions was low, less than or equal to 1.1%. The largest percentages of patients claiming to have moderate problems were observed in the pain/discomfort (40.7%) and anxiety/depression (29.7%) dimensions. The percentage of patients with extreme problems in these dimensions was about 10%.

The levels of reported problems have been categorized into “no problems” (level 1) and “some problem” (level 2 and level 3), according to Table 2. Patients reported problems most frequently in the pain/discomfort (50.7%) and anxiety/depression (38.8%) dimensions. Except for the mobility dimension, statistically significant differences were observed in the other dimensions between patients by geographic regions of the Country (p < 0.05). The South region showed higher percentages of patients with some problem in all dimensions of the EQ-5D-3L. The North region had the smallest percentage of patients reporting a problem in all dimensions, except for the pain/discomfort dimension. The biggest difference between the percentages by region was observed in the anxiety/depression dimension.

**Table 2:** Percentage of patients of the primary health care of the Brazilian Unified Health System who reported no problem or some problem in the dimensions of the EQ-5D-3L, by geographic region. National Survey on Access, Use and Promotion of Rational Use of Medicines – Services, 2015. (n = 8,590)

Dimension of the EQ–5D^a^	North		Northeast		Midwest		South		Southeast		Brazil	
					
	n^b^	% (95%CI)	N	% (95%CI)	n	% (95%CI)	n	% (95%CI)	n	% (95%CI)	n	% (95%CI)
Mobility												p = 0.058
1*	1,349	87.6 (85.7– 89.3)	1,407	83.0 (80.7–85.1)	1,236	83.8 (81.5–85.8)	1,668	81.5 (79.4–83.4)	1,521	84.0 (81.8–85.8)	7,181	83.3 (82.1–84.3)
2	195	12.4 (10.7–14.2)	274	17.0 (14.8–19.3)	280	16.2 (14.5–18.5)	352	18.5 (16.6–20.6)	309	16.0 (14.1–18.1)	1,410	16.7 (15.7–17.8)
Self–care												p = 0.035
1	1,486	96.4 (95.2–97.3)	1,575	93.5 (91.9–94.8)	1,421	94.3 (92.7–95.5)	1,908	93.2 (91.7–94.4)	1,739	95.1 (93.7–96.1)	8,129	94.1 (93.4–94.8)
2	58	3.6 (2.7–4.7)	106	6.5 (5.1–8.1)	95	5.7 (4.5–7.3)	112	6.8 (5.6–8.3)	91	4.9 (3.8–6.2)	462	5.9 (5.2–6.6)
Usual activities												p < 0.001
1	1,398	90.4 (88.6–91.9)	1,405	83.4 (81.1–85.5)	1,267	83.5 (81.2–85.6)	1,713	82.6 (80.6–84.5)	1,585	87.8 (85.9–89.4)	7,368	85.1 (84.0–86.1)
2	146	9.6 (8.1–11.3)	276	16.6 (14.5–18.9)	249	16.5 (14.4–18.7)	306	17.4 (15.5–19.4)	245	12.2 (10.6–14.1)	1,222	14.9 (13.9–15.9)
Pain/discomfort												p < 0.001
1	859	55.1 (52.3–57.7)	724	43.1 (40.2–46.1)	671	48.8 (45.8–51.8)	886	42.4 (39.9–44.9)	1,030	59.0 (56.3–61.6)	4,170	49.3 (47.9–50.8)
2	685	44.9 (42.3–47.6)	957	56.9 (53.9–59.8)	845	51.2 (48.2–54.2)	1,133	57.6 (55.0–60.1)	800	41.0 (38.4–43.7)	4,420	50.7 (49.2–52.1)
Anxiety/depression												p < 0.001
1	1,192	79.2 (77.0–81.3)	977	60.7 (57.8–63.6)	849	56.8 (53.9–59.7)	1,007	51.4 (48.8–53.9)	1,227	66.4 (63.7–68.9)	5,252	61.1 (59.7–62.5)
2	352	20.8 (18.7–22.9)	704	39.3 (36.4–42.2)	667	43.2 (40.2–46.1)	1,012	48.6 (46.0–51.2)	603	33.6 (31.1–36.3)	3,338	38.8 (37.5–40.3)

^a^ 1 (Level 1) – no health problems reported; 2 (Level 2) – some health problem reported (moderate or extreme); ^b^ unweighted n.

Source: PNAUM – Services, 2015.

**Figure 2:**
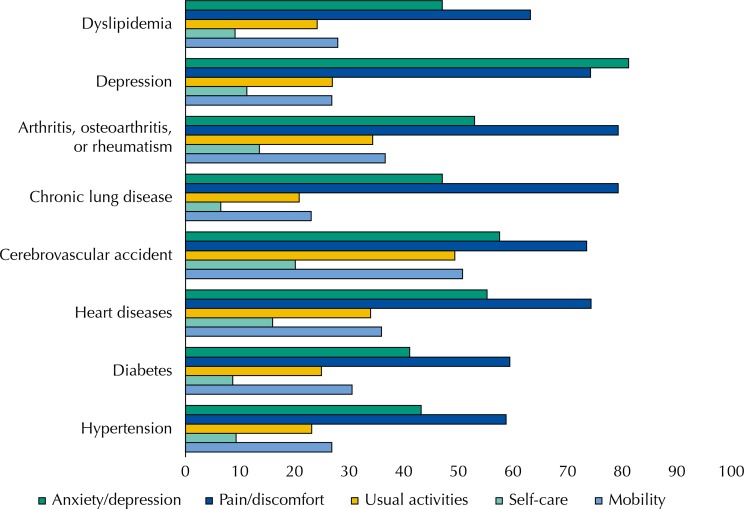
Percentage of patients of the primary health care of the Brazilian Unified Health System with some problem (moderate or extreme) in the dimensions of the EQ-5D-3L, by type of chronic condition. National Survey on Access, Use and Promotion of Rational Use of Medicines – Services, 2015. (n=8,590)

Source: PNAUM – Services, 2015.

In analyzing the descriptive system of the EQ-5D-3L by chronic condition (Figure 2), it was observed that CVA was the chronic condition for which patients most often reported some problem in the self-care, usual activities, and mobility dimensions. In the pain/discomfort dimension, the chronic conditions that most stood out were arthritis, osteoarthritis, and rheumatism. In general, high blood pressure, diabetes, and dyslipidemia had the lowest percentages of “some problem” in the dimensions of the EQ-5D-3L.

Concerning the EQ-5D-3L index of the 8,590 patients of the primary health care of SUS, an average score of 0.793 was observed (95%CI: 0.788–0.799). In the simple linear regression analysis, after the adjustment by region, all the analyzed factors, except skin color and health insurance, were statistically associated with the EQ-5D-3L index (p < 0.05) (data not presented).

Table 3 presents the final model of the multivariate linear regression analysis. The highest deficit in HRQoL was observed for patients who reported having CVA, followed by arthritis, osteoarthritis, or rheumatism. In this model, a negative association (p<0.05) was observed among female patients; patients who reported having CVA, arthritis/osteoarthritis/rheumatism, depression, and heart diseases; patients who reported a poor self-assessment of health; patients who reported drinking once or more per month; and patients who declared dieting to lose weight, to avoid salt intake, and to reduce fat intake.

**Table 3:** Results of the final model of multiple linear regression of independent variables with the EQ-5D-3L index. National Survey on Access, Use and Promotion of Rational Use of Medicines – Services, 2015. (n = 8,374)

Variable	Average index*	Coefficient	95%CI (Coefficient)	p
DEMOGRAPHIC				
Sex				
Male	0.812	-	-	-
Female	0.787	-0.012	(-0.024; -0.000)	0.044
Region of residence				
Midwest	0.793	-	-	-
North	0.846	0.049	(0.037; 0.062)	< 0.001
Northeast	0.777	0.005	(-0.009; 0.018)	0.508
South	0.757	-0.010	(-0.023; 0.035)	0.151
Southeast	0.826	0.032	(0.019; 0.048)	< 0.001
SOCIOECONOMIC				
Education level				
Illiterate	0.720	-	-	-
Some Elementary or Middle School	0.769	0.025	(0.005; 0.044)	0.013
Elementary or Middle School	0.815	0.028	(0.008; 0.048)	0.006
High School	0.839	0.034	(0.014; 0.054)	0.001
Higher Education	0.831	0.028	(-0.006; 0.056)	0.050
HEALTH CONDITION AND LIFESTYLE				
Heart diseases				
No	0.805	-	-	-
Yes	0.667	-0.032	(-0.053; -0.012)	0.002
Cerebrovascular accident				
No	0.799	-	-	-
Yes	0.583	-0.106	(-0.157; -0.055)	< 0.001
Arthritis, osteoarthritis, or rheumatism				
No	0.826	-	-	-
Yes	0.660	-0.086	(-0.099; -0.072)	< 0.001
Depression				
No	0.823	-	-	-
Yes	0.662	-0.079	(-0.092; -0.067)	< 0.001
Self-assessment of health				
Very good/good	0.871	-	-	-
Neither good nor poor	0.718	-0.118	(-0.129; -0.107)	< 0.001
Poor/very poor	0.563	-0.239	(-0.262 −0.215)	< 0.001
Practice of physical activity				
No	0.791	-	-	-
Yes	0.800	0.019	(-0.009; 0.030)	< 0.001
Diet to lose weight				
No	0.802	-	-	-
Yes	0.756	-0.014	(-0.025; -0.002)	0.025
Diet to reduce fat intake				
No	0.827	-	-	-
Yes	0.765	-0.017	(-0.029; -0.005)	0.004
Diet to avoid salt consumption				
No	0.830	-	-	-
Yes	0.767	-0.018	(-0.029; -0.005)	0.003
Alcohol intake				
Never	0.786	-	-	-
Less than once a month or less	0.814	-0.002	(-0.015; 0.011)	0.725
Once or more per month	0.817	-0.020	(-0.020; 0.007)	0.005

* Computed based on the five dimensions of the EQ-5D-3L; R^2^ = 0.3732

Source: PNAUM – Services, 2015.

A positive association was observed (p < 0.05) between HRQoL and patients who lived in the North and Southeast regions, those who reported practicing physical activities, and the increase in educational level. We highlight that, in the multivariate analysis, the variables income and education showed collinearity, and the association between education level and HRQoL prevailed (Table 3).

Variables related to health services did not remain in the final model of the multivariate analysis.

In the multiple linear regression analysis, the index of the EQ-5D-3L and the independent variables explain 37.3% of the model’s variance. The waste did not show any significant pattern that interfered in the validity of the model and showed homoscedasticity.

## DISCUSSION

HRQoL is a subjective and multidimensional measure. More than half of the patients of primary health care in Brazil reported feeling pain or discomfort and nearly half reported being anxious or depressed. Approximately 10% of patients reported pain or extreme discomfort, and being extremely anxious or depressed. The average HRQoL score of the patients was of 0.793, on a scale in which 1 represents the best state of health. The following factors were significantly associated with a worse quality of life: being female; having arthritis, osteoarthritis, or rheumatism; CVA; heart diseases; depression; poor self-assessment of health; drinking alcoholic beverages once or more per month; dieting to lose weight, avoiding salt intake, and reducing fat intake.

Among the five dimensions of the EQ-5D-3L, the prevalence of “some problem” was greater in the pain/discomfort dimension, followed by anxiety/depression and a lower prevalence in self-care. These findings confirm the data from a multicenter study conducted in Brazil with 9,148 individuals^25^ and another study conducted in other Countries^26^.

The prevalence of perfect health in patients, i.e., no problem reported in any of the dimensions of the EQ-5D-3L, was lower than the one observed in the study by Menezes et al.^17^ with 3,363 residents of Minas Gerais. The “perfect health” results obtained were also lower than those observed in international studies, both for the general population and for primary health care^8^ ^,^ ^20^. In relation to the EQ-5D-3L index, the value of the average score found in this study (0.793) was lower than that observed in the study by Menezes et al.^17^ with the general population (0.847). This can be explained by the fact that the patients of this study have been interviewed while waiting to be seen by a primary health care doctor, i.e., they were participants who were seeking health care.

In addition, a high prevalence of chronic conditions was observed in this population (77%) when compared with the one evaluated by Menezes et al.^17^ (50%). Studies have observed higher rates of prevalence of chronic conditions^3^ ^,^ ^28^ and a higher deficit in the HRQoL of primary health care patients in relation to the general population^8^ ^,^ ^20^ ^,^ ^28^.

In line with other studies^3^ ^,^ ^8^ ^,^ ^17^, the highest percentage of patients with some problem was observed for those with CVA; arthritis, osteoarthritis, or rheumatism; and depression. Lung diseases and hypertension had the smallest percentage of patients who reported having some health problem. Cunillera et al.^8^ noted that those with hypertension and diabetes reported a lower percentage of problems when compared to those who reported arthritis. As pointed out by the authors^8^, this may be a reflection of the limited discriminatory capacity of the EQ-5D-3L in detecting moderate problems in certain chronic conditions.

Studies presented a significant negative association between HRQoL and CVA^11^, depression^19^, and heart diseases^1^ ^,^ ^20^. The data of this study were consistent with other national^21^ and international^1^ studies in which arthritis, osteoarthritis, or rheumatism were statistically associated with lower HRQoL scores.

In our study, a significant deficit in the HRQoL was associated with a poor self-assessment of health. This shows a good capability of the EQ-5D-3L to detect health problems in the population. The self-perception of health is a good predictor for mortality and morbidity^23^, and should be a factor to be considered in clinical practice and in health research.

The EQ-5D-3L data of 18 countries showed that age and sex, in smaller proportion, have played important roles in explaining the EQ-5D-3L data among individuals^26^. In this study, a worse HRQoL has been associated with women. The association between a better HRQoL and better socioeconomic conditions is well established in the literature^17^ ^,^ ^18^, and we observed a positive association between HRQoL and increase in educational level.

The profile of inequalities in health in accordance with the dimensions of the EQ-5D-3L has shown different patterns between countries, and pain/discomfort and usual activities were the dimensions that most contributed to these inequalities in most of these countries^26^. In this study, patients from different geographic regions of Brazil showed significant differences both in the dimensions of the descriptive system of the EQ-5D-3L and in association with the EQ-5D-3L index. Patients of the South region showed the highest percentage of “some problem” in all dimensions of the EQ-5D-3L, mainly regarding the anxiety/depression dimension. In addition, a reduction in the HRQoL of the patients of the South region was observed when compared to that of patients in the Midwest, though not significant. Patients of the North and Southeast region showed a significant increase in HRQoL when compared to patients in the Midwest. However, we found no published studies with EQ-5D-3L that allowed comparing these HRQoL data between the regions of the Country.

According to the World Health Organization^29^, diet, physical inactivity, abusive consumption of alcohol and tobacco are important risk factors for the development of chronic conditions. Therefore, the analysis of the association of these factors with HRQoL is fundamental for the monitoring of chronic conditions and the implementation of actions that improve HRQoL. In this study, we observed that patients who practiced physical activities showed a significant positive association with HRQoL. However, we verified a negative statistic association between HRQoL and dieting to lose weight, avoid salt intake, or reduce fat intake. This may be explained by the fact that the dieting advice was given by a doctor or nutritionist and might be related to the possible presence of chronic conditions in these patients. In other population studies, the association between HRQoL and factors such as alcohol consumption, smoking, and physical activity has not been well established yet^10^ ^,^ ^27^.

Variables related to health services were not statistically associated with the HRQoL of patients. Agborsangaya et al.^1^ pointed out that the existence of two or more chronic conditions was associated with reduction in HRQoL, as well as frequent hospitalization and emergency care. However, we found no published studies with EQ-5D-3L that assessed the direct association of these variables with HRQoL.

As health care systems are deliberate social responses to the needs of the population’s health^16^, the primary health care as a closer contact of patients with health care has the potential to intervene in factors influencing HRQoL. The data of our study may allow SUS managers to perform health actions mainly aimed at the dimensions and factors that most affected the HRQoL of primary health care patients. Thus, effective public policies and programs in SUS are needed for ensuring a better HRQoL to its patients. There must be an intersectoral action that encourages the promotion of health in specific communities and population groups, aiming to promote behavior changes and healthy lifestyles^6^ ^,^ ^15^. In addition, there is a need for interventions in the socioeconomic, environmental, and cultural conditions, such as high-quality education^6^. We highlight that not only the health care systems, but patients themselves have a key role in HRQoL and its associated factors. Therefore, it is necessary to promote the autonomy of subjects and groups for ensuring their right to health and HRQoL^15^.

This study was carried out with a representative sample of patients of the primary health care of SUS. However, the observed HRQoL of SUS patients may not necessarily reflect the HRQoL of the general population. Lee et al.^13^ emphasize that the sample may underrepresent less frequent patients. Therefore, the extrapolation of data of patients of the primary health care of SUS to the general population should be performed with caution.

The results of this study show that the HRQoL of patients of primary health care was influenced by demographic and socioeconomic factors, related to health conditions and lifestyle, but not by factors related to health services. Thus, the measurement of the HRQoL of patients of the primary health care of SUScontributes not only to better understand HRQoL and its associated factors, but can also be an important measure to guide actions of health promotion and comprehensive health care to SUS patients. We emphasize that longitudinal studies are needed to determine the causal association between HRQoL and its associated factors.

## Footnotes

a Associação Brasileira de Empresas de Pesquisa. Critério de classificação econômica Brasil: alterações na aplicação do Critério Brasil, válida a partir de 01/01/2014. São Paulo: ABEP; 2014 [cited 2016 Mar 1]. Available from: www.abep.org/Servicos/Download.aspx?id=01
